# Using mHealth to Predict Noncommunicable Diseases: A Public Health Opportunity for Low- and Middle-Income Countries

**DOI:** 10.2196/jmir.7593

**Published:** 2017-05-05

**Authors:** Ellen Rosskam, Adnan A Hyder

**Affiliations:** ^1^ ER Global Consult La Croix-de-Rozon Switzerland; ^2^ Visiting Professor School of Health Sciences University of Massachusetts Lowell, MA United States; ^3^ Bloomberg School of Public Health Department of International Health, Health Systems Program Johns Hopkins University Baltimore, MD United States; ^4^ Bloomberg School of Public Health International Injury Research Unit Johns Hopkins University Baltimore, MD United States

**Keywords:** mHealth, low- and middle-income countries, noncommunicable diseases, research agenda, population health surveys

Nearly 70% of the 56 million deaths that took place globally in 2012 were due to noncommunicable diseases (NCDs), in particular, cardiovascular diseases, cancers, chronic respiratory diseases, and diabetes, and nearly two-thirds of all NCD deaths took place in low- and middle-income countries (LMICs) [1]. If effective steps are not taken to curb the epidemic, deaths due to NCDs are projected to rise exponentially in the coming decade [2]. Key risk factors responsible for a majority of NCDs include tobacco use, unhealthy diet, sedentary lifestyle, and excessive use of alcohol. With targeted action, these behavioral risk factors have demonstrated potential to be modified [3] to reduce NCDs and improve population health. Reducing NCDs, particularly in the world’s poorest countries, can lead to increases in equity and socioeconomic development while reducing poverty due to ill health and promoting sustainable development and social justice.

Key to global efforts to prevent and control NCDs is national surveillance. A promising approach increasingly being explored for public health surveillance involves mobile phones. A nascent yet emergent field, mHealth, describes medical and public health activities that leverage the global proliferation of cellular networks and mobile phone ownership or access to improve population health outcomes. There are nearly 7.5 billion wireless phone subscriptions globally, with the majority (78%) in LMICs [4]. Global connectivity to cellular networks can make large proportions of a population accessible through their mobile phones. In response to the increasing NCD disease burden, the intersecting need for NCD data in LMICs and the near-universal population access to mobile phones in a growing number of countries presents an opportunity for public health.

This special Theme Issue of JMIR offers a step forward in documenting what is known about surveillance of risk factors for NCDs in LMICs using mobile phone surveys (MPS). The evidence illustrates that the state-of-the-art is sufficient to roll out population-level surveys in LMICs using mobile phone platforms while paying careful attention to issues such as ethics, methodology, and turning results into practice. The results offer guidance for policy and practice.

The article, “Noncommunicable Disease Risk Factors and Mobile Phones: A Proposed Research Agenda,” proposes a research and development agenda for NCD risk factors and MPS [5]. The goal of the proposed agenda is to help standardize operating procedures for MPS, which will allow for comparisons of NCD risk factors within and across sites and over time. The potential is explored for MPS to collect such data, review key research issues, and introduce a multicountry effort that seeks to partly respond to this public health challenge. It is hoped that the proposed research agenda will catalyze a global dialogue and action to enhance the use of MPS for NCDs and potentially other public health risk factor surveillance.

Limited evidence exists on the comparative effectiveness of MPS modalities in LMICs although a variety of options are available. “Mobile Phone Surveys for Collecting Population-Level Estimates in Low- and Middle-Income Countries: A Literature Review” reviews the current landscape of MPS being used for population-level data collection in LMICs, specifically through the use of short message service, interactive voice response (IVR), and computer-assisted telephone interview survey modalities [6]. From the articles identified of MPS use to collect population estimates across a range of topics, results reveal that the state of MPS to collect population-level estimates of health and other indicators is a nascent field, indicating the need for more research.

The methodological approach used to test the use of MPS for NCDs is described in “Evaluation of Mechanisms to Improve Performance of Mobile Phone Surveys: A Research Protocol” [7]. Using microtrials, a set of future studies that will help enhance the efficiency and technical effectiveness of MPS is proposed for LMICs. The authors assess the effect of factors such as incentive timing and structure, survey introduction characteristics, different sampling frames, and survey modality on key survey metrics such as survey response, completion, and attrition rates.

Further investigating the literature, “Building the Evidence Base for Remote Data Collection in Low- and Middle-Income Countries: Comparing Reliability and Accuracy Across Survey Modalities” reviews findings that compare a mode of remote data collection to at least one other mode [8]. The synthesis examines MPS mode effects on the reliability and accuracy of results. Findings show, for example, that remote data collection consistently elicited higher reports of socially nondesirable behaviors compared to in-person data collection. The review reveals the need for additional studies that compare reliability and construct validity across survey modalities.

IVR has the potential to expand current surveillance coverage and data collection. Two rounds of IVR pilot testing in Baltimore, Maryland, revealed that most participants felt this type of survey would lead to more honest, accurate responses than face-to-face questionnaires, especially for sensitive topics. In the pilot tests, participants indicated a clear comprehension of the IVR-administered questionnaire and that the IVR platform was user-friendly. Described in “The Development of an Interactive Voice Response Survey for Noncommunicable Disease Risk Factor Estimation: Technical Assessment and Cognitive Testing,” the authors conclude that formative research and cognitive testing of the questionnaire are needed for deployment in LMICs [9].

The near-ubiquitous ownership of phones in LMICs, high population mobility, and low cost demand a reexamination of statistical recommendations for MPS, especially when surveys are automated. In “Health surveys using mobile phones in developing countries: automated active strata monitoring and other statistical considerations for improving precision and reducing biases,” methods are proposed to reduce estimate bias and to adjust for selectivity due to mobile ownership [10]. The authors describe using automated active strata monitoring (AASM) to improve representativeness of the sample distribution to that of the source population. They conclude that although some statistical challenges remain, MPS represents a promising emerging means for population-level data collection in LMICs.

The increasing use of MPS in LMICs brings forth a cluster of ethical challenges. The existing literature regarding the ethics of mobile or digital health, however, mainly focuses on the use of technologies in high-income countries and does not consider the specific ethical issues associated with the conduct of MPS for NCD risk factor surveillance in LMICs. In “Ethics Considerations in Global Mobile Phone-Based Surveys of Noncommunicable Diseases: A Conceptual Exploration,” the authors explored central ethics issues in this domain, including identifying the nature of the activity, stakeholder engagement, appropriate design, anticipating and managing potential harms and benefits, consent, reaching intended respondents, data ownership, access and use, and ensuring LMIC sustainability [11]. The authors call for future work to develop a broad conceptual framework for the ethical, legal, and societal issues associated with MPS for NCD risk factors. They further point to the need for guidance documents to identify key issues, outline pros and cons of options available to stakeholders for each issue, review additional points to consider, and provide references to resources relevant to each issue. In order to begin to address the various needs, the researchers hope to establish a global working group inclusive of experts in ethics, mHealth survey implementation, regulatory oversight and policy, public health, social science, and MPS platform development.

The article, “Moving the Agenda on Noncommunicable Diseases: Policy Implications of Mobile Phone Surveys in Low- and Middle-Income Countries,” presents the special challenges for policy makers [12]. The article discusses potential benefits of MPS for developing, implementing, and evaluating NCD prevention and control policies. It includes an overview of major global commitments to NCD prevention and control as well as an exploration of how countries can translate these commitments into policy action at the national level. Potential benefits of MPS are discussed, including cost benefits of MPS for informing NCD policy actions compared to using traditional household surveys, timeliness of assessments to feed into policy and planning cycles, tracking progress of interventions, timely course correction for suboptimal or noneffective interventions, and assessing fairness in financial contribution and financial risk protection for those affected by NCDs in the spirit of universal health coverage, inter alia. The authors demonstrate how MPS can become a powerful tool for collecting population-based data to inform policies that address key public health challenges such as NCDs. Further research in real-life settings will help to provide additional realistic world experiences.

This special issue of JMIR offers a step forward in benchmarking what is known and what is possible to know using MPS for data collection and surveillance systems. These results offer guidance for research expectations and opportunities to understand and curb the rise of NCDs in LMICs. Additional next steps are foreseen to continue documenting empirical experiences of MPS use in LMICs to collect risk factor data on NCDs, engaging with global bodies toward the development of a research agenda, establishing a global working group of experts to address the ethical issues surrounding MPS use in LMICs, and working with international and national level policy-makers to create a comparative framework for turning results into policy and practice.

## Abbreviations

IVR: interactive voice response
LMIC: low- and middle-income country
MPS: mobile phone surveys
NCD: noncommunicable disease
CATI: computer-assisted telephone interview
SMS: short message service
